# The Effectiveness of Smartphone Apps for Lifestyle Improvement in Noncommunicable Diseases: Systematic Review and Meta-Analyses

**DOI:** 10.2196/jmir.9751

**Published:** 2018-05-04

**Authors:** Pernille Lunde, Birgitta Blakstad Nilsson, Astrid Bergland, Kari Jorunn Kværner, Asta Bye

**Affiliations:** ^1^ Department of Physiotherapy Faculty of Health Sciences OsloMet—Oslo Metropolitan University Oslo Norway; ^2^ Section for Physiotherapy Division of Medicine Oslo University Hospital Oslo Norway; ^3^ Center for Connected Care Oslo University Hospital Oslo Norway; ^4^ Department of Strategy and Entrepreneurship BI Norwegian School of Business Oslo Norway; ^5^ Department of Nursing and Health Promotion Faculty of Health Sciences OsloMet—Oslo Metropolitan University Oslo Norway; ^6^ European Palliative Care Research Centre Department of Oncology Oslo University Hospital and Institute of Clinical Medicine, University of Oslo Oslo Norway

**Keywords:** smartphone, telemedicine, noncommunicable diseases, lifestyle, diet, exercise

## Abstract

**Background:**

Noncommunicable diseases (NCDs) account for 70% of all deaths in a year globally. The four main NCDs are cardiovascular diseases, cancers, chronic pulmonary diseases, and diabetes mellitus. Fifty percent of persons with NCD do not adhere to prescribed treatment; in fact, adherence to lifestyle interventions is especially considered as a major challenge. Smartphone apps permit structured monitoring of health parameters, as well as the opportunity to receive feedback.

**Objective:**

The aim of this study was to review and assess the effectiveness of app-based interventions, lasting at least 3 months, to promote lifestyle changes in patients with NCDs.

**Methods:**

In February 2017, a literature search in five databases (EMBASE, MEDLINE, CINAHL, Academic Research Premier, and Cochrane Reviews and Trials) was conducted. Inclusion criteria was quantitative study designs including randomized and nonrandomized controlled trials that included patients aged 18 years and older diagnosed with any of the four main NCDs. Lifestyle outcomes were physical activity, physical fitness, modification of dietary habits, and quality of life. All included studies were assessed for risk of bias using the Cochrane Collaboration`s risk of bias tool. Meta-analyses were conducted for one of the outcomes (glycated hemoglobin, HbA_1c_) by using the estimate of effect of mean post treatment with SD or CI. Heterogeneity was tested using the I^2^ test. All studies included in the meta-analyses were graded.

**Results:**

Of the 1588 records examined, 9 met the predefined criteria. Seven studies included diabetes patients only, one study included heart patients only, and another study included both diabetes and heart patients. Statistical significant effect was shown in HbA_1c_ in 5 of 8 studies, as well in body weight in one of 5 studies and in waist circumference in one of 3 studies evaluating these outcomes. Seven of the included studies were included in the meta-analyses and demonstrated significantly overall effect on HbA_1c_ on a short term (3-6 months; *P*=.02) with low heterogeneity (I^2^=41%). In the long term (10-12 months), the overall effect on HbA_1c_ was statistical significant (*P*=.009) and without heterogeneity (I^2^=0%). The quality of evidence according to Grading of Recommendations Assessment, Development and Evaluation was low for short term and moderate for long term.

**Conclusions:**

Our review demonstrated limited research of the use of smartphone apps for NCDs other than diabetes with a follow-up of at least 3 months. For diabetes, the use of apps seems to improve lifestyle factors, especially to decrease HbA_1c_. More research with long-term follow-up should be performed to assess the effect of smartphone apps for NCDs other than diabetes.

## Introduction

Noncommunicable diseases (NCDs) account for as much as 70% of all deaths globally [1]. The four main NCDs are cardiovascular diseases (CVDs), cancers, chronic pulmonary diseases, and diabetes mellitus (DM), which all share the same behavioral risk factors: physical inactivity, unhealthy diet, tobacco use, and harmful use of alcohol [1]. Lifestyle changes toward a more healthy behavior are of great importance in both prevention and treatment of these NCDs [2-5].

Adherence to treatment is the most important modifiable factor that compromises treatment outcome. Traditionally, adherence has focused on medication, which also is reflected in the World Health Organization’s (WHO) definition of adherence; “the extent to which the patient follows medical instructions” [6]. However, adherence also encompasses numerous health-related behaviors such as smoking cessation and changes in physical activity (PA), exercise, or diet, which are considered as a major challenge in treatment of NCDs [6]. Usually the interventions designed to promote healthy behavior are conducted as face-to-face modes of delivery, and their mainly short-term effectiveness has been extensively documented in a number of systematic reviews [7-10]. One reason of the inconclusive long-term results are probably lack of systematic follow-up and monitoring, which are crucial elements of all effective health behavior change [11].

Feedback seems to be essential for success in behavioral change [12]. Modern technology such as electronic devices permits structured monitoring of important health parameters and follow-up of patients with NCD [13]. A meta-analyses (n=43,200) documented that mixed mode of delivery interventions where traditional behavioral change techniques (BCTs) were used together with dedicated digital tools were more effective than traditional techniques for behavioral change alone [12]. Another meta-analyses (n=20,000) supports this and concludes that tailored Web-based interventions was significantly more effective in improving health outcomes compared with nontailored Web-based interventions [14]. Although several interventions such as Web portals, SMS text messaging (short message service, SMS), and phone calls to improve health for patients with NCDs are promising [15-19], smartphone technology has been emphasized because of its possibility to monitor and follow-up patients’ health from anywhere at any time [20].

Thus, the purpose of this systematic review was to examine the effectiveness of interventions with smartphone apps, lasting at least 3 months, to promote lifestyle changes such as PA, physical fitness, modification of dietary habits, and quality of life (QoL) in patients with NCDs.

## Methods

### Reporting Standards

This systematic review and its procedures were planned, conducted, and reported in accordance to the Preferred Reporting Items for Systematic Reviews and Meta-Analyses (PRISMA) guidance. The review protocol was registered in the International Prospective Register of Systematic Reviews, registration number CRD42017057796.

### Inclusion Criteria

Randomized and nonrandomized clinical trials with a minimum of 3-months follow-up that evaluated the effect of interventions with apps aiming to monitor PA and/or dietary habits were considered for inclusion. Patients had to be aged 18 years or above and diagnosed with CVD, cancer, chronic pulmonary disease, or DM. Case series with 10 or less participants were not included. If change of lifestyle was not the main goal of the intervention, studies were excluded. Due to limited resources for translation, the review was restricted to publications in Norwegian and English.

### Outcome Measures

Primary outcomes of interest were PA, physical fitness, modification of dietary habits, and/or QoL. Regarding PA and physical fitness, the following measures were considered relevant: steps, self-reported minutes in activity, self-reported minutes of exercise, maximal oxygen consumption, 6-min walk test, shuttle walk tests, and submaximal physical fitness tests. Regarding effect on modification of dietary habits, measures included body weight, body mass index (BMI), waist circumference, and glycated hemoglobin (HbA_1c_). Both generic and disease-specific QoL questionnaires were evaluated.

### Search Strategy

Five databases (EMBASE, MEDLINE, CINAHL, Academic Research Premier, and Cochrane Reviews and Trials) were systematically searched for relevant studies with help from a research librarian. Boolean operators were used to expand, exclude, or join keywords in the search using the terms “AND” and “OR.” Articles published before February 23, 2017 in English were included in this systematic review. The search strategy of each database is listed in Multimedia Appendix 1.

### Selection of Studies

**Figure 1:**
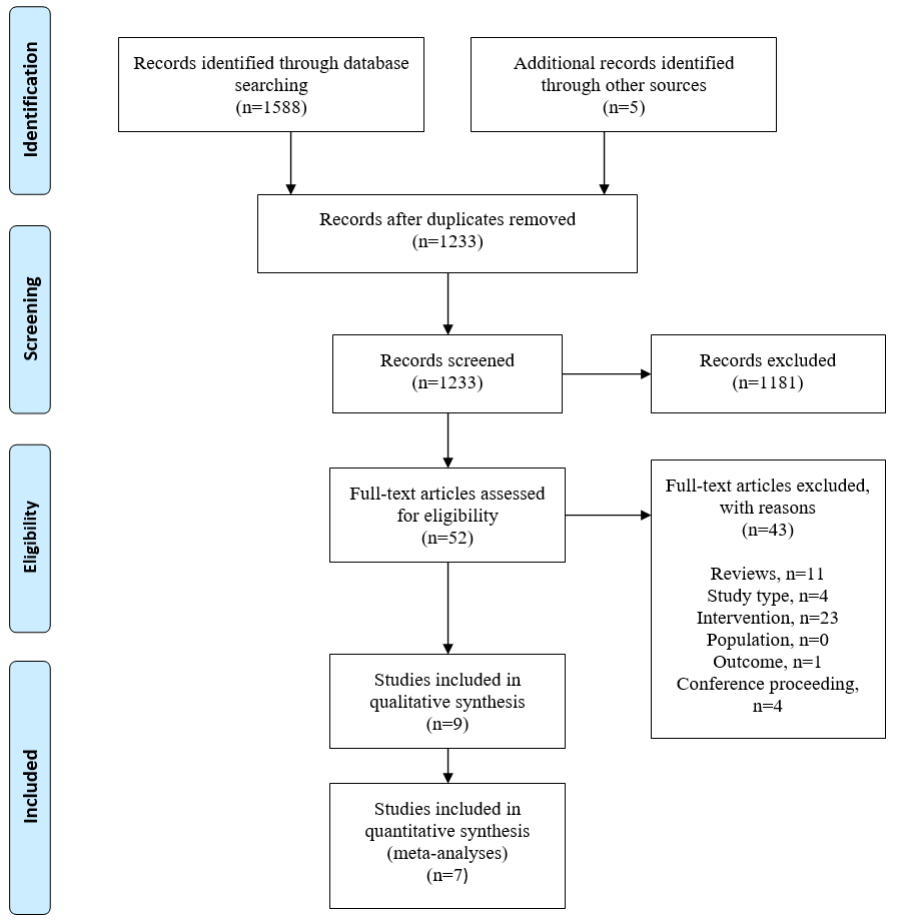
Preferred Reporting Items for Systematic Reviews and Meta-Analyses (PRISMA) flow diagram of reviewed and included studies.

Figure 1 shows the PRISMA flowchart of reviewed and included studies.

The first author conducted the database search assisted by a research librarian. After conducting the search, duplicates were removed, and 2 authors independently reviewed title and abstract of all studies. We kept relevant reviews to hand screen the reference lists in case some articles got lost in the initial search. Disagreements between the two authors conducting the title and abstract review were discussed until a consensus was reached. All the studies that met the inclusion criteria went through a full-text screening process by two reading pairs. The first author reviewed all the studies. In the full-text screening phase, we hand screened the reference lists of all reviews, and we also screened the study characteristics of the included studies in the reviews. Additional studies were identified for inclusion to full-text screening. In case of disagreement in this phase, the other reading pair contributes to achieve consensus. In case of uncertainty related to the intervention used in some of the studies, we contacted authors. In addition, phone developers’ own description was used if there was any uncertainty whether the phones were smartphones or not.

The first author extracted data from the studies. In studies with more than one intervention arm, data from the most intervening arm were extracted [21,22]. This was done to make the interventions in the different studies as homogeneous as possible. Data extracted from the studies included authors, year, country, study design, patient group (sample size and disease), inclusion criteria, details of the interventions, outcomes, and results.

**Figure 2:**
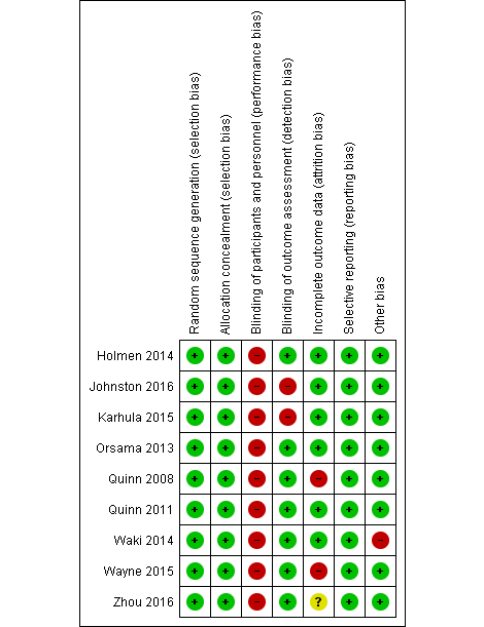
Risk of bias within studies.

### Quality Assessment

In total, five reviewers (two reading pairs and first author reading all papers) independently assessed each included study for risk of bias (high, low, or unclear) using the Cochrane Collaboration`s risk of bias tool [23]. Again, the other reading pair assisted to reach consensus if necessary. Regarding scoring the different studies with respect to “selective reporting,” we followed the judging criteria given by Cochrane Collaboration and read all protocols available in clinical trials or in journals if published. The results of the risk of bias assessment were then exported to the software RevMan, version 5.3 [24] to create visual representation of the publication (Figure 2). Difficulty in scoring some of the studies was handled by reading the protocol if published, either in paper or in Clinical Trials and/or by contacting study authors.

### Data Synthesis and Statistical Analyses

After the first author had extracted data from the studies, all authors evaluated the preliminary results of the review. Meta-analyses were performed based on sufficient homogeneity across most of the included studies with regard to disease (DM) and primary outcome (HbA_1c_). One meta-analysis for short-term effect (3-6 months) and one for long-term effect (10-12 months) were performed. In the end, all studies included in the meta-analyses were evaluated using the Grading of Recommendations Assessment, Development, and Evaluation (GRADE) [25].

Meta-analyses were conducted by using estimate of effect of mean posttreatment HbA_1c_ values for both intervention and control group with SD. In studies where mean change were the only presented result [26,27], we used this in addition to SD for both groups. If only CIs were presented, we calculated SD. In one study [27], both adjusted and nonadjusted estimate of effect were presented. We chose to use the adjusted estimate as authors reported this as results. To carry out the meta-analyses, we pooled studies based on length of the interventions. Heterogeneity was tested using the I^2^ test. Forest plot were constructed to visualize the results. All analyses were performed with RevMan version 5.3 software, with double entry of the estimate of effect.

## Results

### Study Selection

Our search results are summarized in the PRISMA flow diagram (Figure 1). A total of 1588 studies were identified. Duplicates were removed, leaving 1228 studies to screen. After screening title and abstracts, 1181 papers were excluded for not meeting the inclusion criteria, leaving 47 studies for full-text review. On the basis of the full-text review, 6 studies were included in this review. From a hand search of review paper references and study characteristics in the reviews, an additional 5 studies were identified as potentially eligible, of which 3 studies were included. In total, 9 studies were included in the systematic review and 8 were eligible for meta-analysis [21,22,26-31]. One study [28] was not included in the meta-analysis because of poor outcome reporting and lack of response on email.

### Study Characteristics

The characteristics of the included studies are presented in Table 1. Six of the included studies included patients with DM type 2 only [21,22,27-30]. One study included both type 1 and type 2 DM [31], 1 study included patients with DM type 2 or CVD (ischemic heart disease and/or heart failure) [26], and 1 study included patients with myocardial infarction [32]. Four studies were carried out in Europe [21,26,27,32], 3 in North- and East America [22,28,30], and 2 in Asia [29,31]. Study duration ranged from 3 months to 1 year of follow up; 3 months (n=3) [28,29,31], 6 months (n=2) [30,32], 10 months (n=1) [27], and 1 year (n=3) [21,22,26]. All the included studies had a control group, where 6 studies involved “usual care” or “standard medical care” as control. Two of the included studies gave the control group part of the intervention, whereas one of them received a simplified smartphone app with drug adherence e-diary [32], and the other received health coaching [30]. One study did not describe what the control group received [28].

### Intervention Characteristics

An overview of the characteristics of the interventions used in included studies is presented in Table 2. All the included studies [21,22,26-32] used apps where it was possible to register blood glucose data. All studies had registration of lifestyle factors, whereas 6 studies registered exercise and/or PA in the app [21,26,27,29,30,32] and/or registration of dietary habits [21,22,28-31]. Patients in all studies monitored themselves on lifestyle factors and clinical measurements. In 7 of the studies [22,26-31], health personal and/or researchers monitored them as well. Patients received feedback based on what they had registered in the app, whereas 4 of the studies had automatic feedback [21,22,28,32], 3 studies had individualized feedback [26,30,31], 1 study had automatic feedback and individualized if warranted [27], and 1 study had both automatic and individualized [29]. In 6 of the included studies, they had additional support to the app [21,22,26-28,30]; however, the app was the main part of the intervention.

### Risk of Bias Within Studies

There was high risk of bias in all the included studies (Figure 2).

Six of the studies were registered in clinical trials [21,22,26,30-32]. All of them reported on more outcomes than registered. The additional outcomes were not exclusively positive. The reason might be not updating the study protocol rather than selective outcome reporting. Therefore, they got “low risk of bias” on “selective reporting” score. The three other studies were neither registered in clinical trials nor published elsewhere [27-29]. However, the published reports included all expected outcomes, and therefore, they were all given “low risk of bias.”

### Effects of Smartphone App

An overview of effects of apps on lifestyle factors including physical fitness, PA, modification of dietary habits, and QoL is presented Table 1. Five of 8 studies evaluating HbA_1c_ reported statistical significant differences between groups in favor of the intervention groups [22,27-29,31]. One of 3 studies evaluating waist circumference reported a statistical significant effect between groups in favor of the intervention group [26]. Additionally, 1 study reported a statistical significant within group change for the intervention group [30]. One of 5 studies evaluating body weight reported statistical significant differences between groups in favor of the intervention group [27], and 2 studies reported a statistical significant change in body weight within the intervention groups [26,30].

**Table 1:** Study characteristics.

Reference (year), country	Study design and study duration	Sample size; disease	Intervention group (IG) or control group (CG)	Outcomes of interest^a^	Results^b^
Holmen et al (2014), Norway [21]	3-arm randomized controlled trial (RCT), multicenter, 12 months	N=151; Diabetes mellitus (DM) type 2	IG 1: app to increase self-management ; IG 2: IG 1 + five health counseling sessions by a diabetes nurse; CG: usual care	*Glycated hemoglobin (HbA_1c_)*, Weight, Health-related quality of life (HRQoL; 36-item short form survey, SF-36), Lifestyle change (dietary and physical activity)	No statistical differences between groups (NS) in outcomes of interest
Johnston et al (2016), Sweden [32]	2-arm RCT, multicenter, 6 months	N=174; Myocardial infarction	IG: app to register information about drug adherence, exercise, weight, smoking, blood pressure, low-density lipoprotein cholesterol, and blood glucose; CG: simplified app with drug adherence e-diary	Cardiovascular risk (body mass index, physical activity), QoL (EuroQoL-5D)	NS in outcomes of interest
Karhula et al (2015), Finland [26]	2-arm RCT, 12 months	N=519; Heart disease patients (ischemic and/or heart failure) or DM type 2	IG: app with health coaching and self-monitoring of health parameters; CG: usual care	*HRQoL (SF-36), HbA_1c_ (in DM patients),* Body weight, Waist circumference	Diabetics: Change in waist circumference, *P*=.01; IG: −2.03, 95% CI (−2.76 to −1.29), CG: − 0.29, 95% CI (−1.47 to 0.9); NS in other outcomes of interest; Heart patients: NS in all outcomes of interest
Orsama et al (2013), Finland [27]	2-arm RCT, 10 months	N=53; DM type 2	IG: app for monitoring and remote reporting of diabetes health-related parameters; CG: usual care	*HbA_1c_,* Body weight	Change in HbA_1c_, *P*=.02, IG: −0.4, 95% CI (−0.67 to −0.14), CG: 0.004, 95% CI (−0.35 to 0.36)-Change in body weight, *P*=.02, IG: −2.1 kg, 95% CI (−3.6 to −0.6), CG: 0.4 kg, 95% CI (−1.1 to 1.9)
Quinn et al (2008), Maryland, United States [28]	2-arm RCT, multicenter, 3 months	N=30; DM type 2	IG: app with monitoring of health parameters; CG: not mentioned	*HbA_1c_*	Change in HbA_1c_, *P*=.04, IG: −2.03%, CG: −0.68%
Quinn et al (2011), Maryland, United States [22]	4-arm cluster RCT, 12 months	N=163; DM type 2	IG 1: app allowing patients to enter diabetes self-care data. Web portal that augmented the app. Health providers had access to analyzed patient data; IG 2: as IG 1, but in the Web portal, health providers had access to unanalyzed patient data; IG 3: as IG 2, but the health providers had only access to patient data if the patients chose to share it; CG: usual care	*HbA_1c_*	Change in HbA_1c_, *P*=.001, 95% CI change in IG: −2.3 to −1.5, CG: −1.1 to −0.3
Waki et al (2014), Japan [29]	2-arm RCT, 3 months	N=54; DM type 2	IG: app aiming to increase self-management; CG: usual care, continue their self-care regimen	*HbA_1c_,* Body mass index (BMI)	Change in HbA_1c_, *P*=.015, IG: −0.4%, CG: 0.1%; NS in other outcomes of interest
Wayne et al (2015), Canada [30]	2-arm RCT, multicenter, 6 months	N=131; DM type 2	IG: app monitoring health parameters; CG: usual care and health coaching	*HbA_1c_,* Body weight, BMI, Waist circumference, QoL (SF-12)	NS in outcomes of interest.
Zhou et al (2016), China [31]	2-arm RCT, 3 months	N=100; DM type 1 and type 2	IG: app monitoring health parameters; CG: usual care	*HbA*_1c,_ Body weight, BMI, Waist circumference	Change in HbA_1c_, *P*<.01, IG: −1.95%, CG: −0.79%; NS in other outcomes of interest.

^a^Outcome in italics indicate primary outcome in the study.

^b^Results are reported as difference between groups (*P* value) and as mean change in each group in accordance what is used by the authors.

**Table 2:** Intervention characteristics.

Smartphone app						Additional support^a^
First author (year)	Logging lifestyle factors	Clinical measurements logging	Monitoring personnel	Education or information	Feedback	
Holmen et al (2014) [21]	✓^b^	Blood glucose (BG)	Patient	✓	Automatic	✓ (1,3)
Johnston et al (2016) [32]	✓	Blood pressure (BP), BG, Low-density lipoprotein cholesterol, Weight	Patient	✓	Automatic	
Karhula et al (2015) [26]	✓	BP, Weight, BG (diabetics)	Patient, Health-coach	✓	Individualized via telephone every 4-6 weeks	✓ (2,3)
Orsama et al (2013) [27]	✓	BP, Weight, BG	Patient, Study nurses	✓	Automatic, Individualized if warranted	✓ (2)
Quinn et al (2008) [28]	✓	BG	Research team, Patient, Physician	✓	Automatic	✓ (2,4)
Quinn et al (2011) [22]	✓	BG	Patient, Health care provider	✓	Automatic	✓ (2,3)
Waki et al (2014) [29]	✓	BG, BP, Weight	Patient, Research team, Dietitian		Automatic, Individualized	
Wayne et al (2015) [30]	✓	BG, Mood	Patient, Health coach		Individualized	✓ (1,3)
Zhou et al (2016) [31]	✓	BG, BP	Patient, Research team	✓	Individualized	

^a^1: Exercise advice; 2: Patient Web portal; 3: Telephone contact or coaching; 4: Email.

^b^Check mark denotes characteristic is present.

### Effect of Smartphone App for Patients With Diabetes With Regard to Glycated Hemoglobin

**Figure 3:**
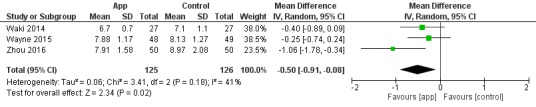
Forest plot: short-term effect on glycated hemoglobin (HbA1c).

**Figure 4:**
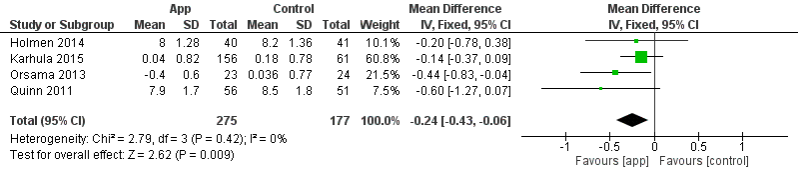
Forest plot: long-term effect on glycated hemoglobin (HbA_1c_).

Seven studies were included in the quantitative synthesis; 3 studies evaluated the effect of apps on short term [29-31], and 4 studies on long term [21,22,26,27]. The overall effect on short term was statistically significant (*P*=.02; Figure 3). The heterogeneity was acceptable with I^2^ at 41%. The overall effect on long term was statistically significant (*P*=.009) with no heterogeneity (I^2^=0%; Figure 4).

The quality of evidence (GRADE) is presented in Table 3. The quality of evidence in the included studies in short- and long-term effect analysis was scored as low and moderate, respectively. In the short-term effect analysis, the quality was downgraded because of risk of bias and imprecision [29-31]. In the long-term effect analysis, the quality was downgraded to moderate because of imprecision in the estimate of effect [21,22,26,27].

**Table 3:** Quality of evidence of glycated hemoglobin (HbA_1c_).

Outcome	Number of participants (number of studies)	Standardized mean differences (95% CI)	Quality of evidence (GRADE^a^)
HbA_1c_ short term	251 (3)	−0.50 (−0.91 to −0.08)	Low^b,c^
HbA_1c_ long term	452 (4)	−0.24 (−0.43 to −0.06)	Moderate^d^

^a^GRADE: Grading of Recommendations Assessment, Development, and Evaluation.

^b^Downgraded because of risks of biases (such as attrition bias, blinding, and other bias).

^c^Downgraded because of imprecision (few participants, less than 300).

^d^Downgraded because of imprecision (variation in the estimate of effect).

## Discussion

This is, to our knowledge, the first systematic review that examines the effectiveness, for at least 3 months, of apps to promote lifestyle changes for patients with NCD. Eight of 9 studies in this review were performed in persons with DM. In this group, the app showed better effectiveness to improve lifestyle factors than traditional ways to intervene and/or no intervention, especially regarding decrease of HbA_1c_. Only 2 studies had included persons with CVD, and no differences were found in variables reflecting lifestyle.

A major strength of this review are the authors’ attempt to identify all relevant studies by using a comprehensive search strategy in multiple databases led by a research Liberian, as well as well documented methodological strictness performing the systematic review and meta-analyses. In total, five authors participated in this process, which also included hand searching of review paper references to identify additional studies that may have been lost in the initial search. All authors also independently screened the studies for risk of bias. As the search results turned out to be relatively homogeneous, it also was possible to pool the results of one common outcome into two meta-analyses and grade them. However, despite the existence of hundreds of studies involving apps used by CVD, cancer, chronic pulmonary, and/or DM patients, there is a lack of rigorous trials regarding specific lifestyle outcomes such as PA, physical fitness, modification of dietary habits, and QoL.

Statistical significant improvements between groups on lifestyle factors were reported in 6 of 9 studies (67%). To our knowledge, only one systematic review has previously evaluated the impact of mobile health (mHealth), which WHO has defined as medical and public health practice supported by mobile devices such as mobile phones patient monitoring devices and other wireless devices [33], in more than one chronic disease. They reported significant improvements between groups on disease-specific outcomes in 39% of the 41 included studies [34]. The different results may be explained by different study aims. Although the aim of this review was to study the effectiveness of apps to promote lifestyle changes, the former review aimed to assess the usability, feasibility, and acceptability of mHealth interventions. It is therefore likely that the included studies [34] also were designed to assess usability, feasibility, and acceptability and not necessarily to improve lifestyle and disease-specific outcomes.

To our knowledge, only one systematic review and meta-analyses on the effect of apps to improve HbA_1c_ has previously been conducted [35]. This review included several studies also included in this review [21,22,27-29], but they did not have any exclusion criteria based on follow-up, and the results were pooled into meta-analyses based on methodological quality. They reported a mean reduction in HbA_1c_ in participants using an app compared with control of 0.49% (95% CI 0.3-0.68; I^2^=10%). Studies with fair or good quality showed lower effect compared with studies with poor quality [35]. In the current meta-analyses, the mean reduction in HbA_1c_ in participants using an app compared with controls were 0.50% (95% CI 0.08-0.91; I^2^=41%) and 0.24% (95% CI 0.06-0.43; I^2^=0%) for short term and long term, respectively.

Despite the fact that the majority of the included studies showed significant efficacy, 3 of the included studies [21,30,32] did not show any effect on outcomes of interest, and significant effect were not found in health-related QoL [21,26,30,32]. One explanation for this might be the fact that the studies did not have enough power to detect such differences, as HbA_1c_ was the primary outcome, and statistical power and the intervention design were based on this. In addition, we should not ignore the fact that it might be with apps similar to other lifestyle interventions, it is hard to actually get a change that lasts over time [6]. This may be what we see as a tendency in our meta-analyses regarding HbA_1c_ as well, where short-term effect is superior to long-term effect.

A recent systematic meta-review evaluated telehealth interventions, which are also regarded as mHealth, to support self-management of long-term conditions [36]. It revealed that most of the research in the field of technology-based interventions is currently conducted in patients with DM, and their results support our findings. Monitoring of blood glucose and feedback improved glycemic control in patients with DM [36]. Meta-analysis on the effects of mHealth in patients with DM have reported a significant reduction in HbA_1c_ of 0.33% [37]. Such interventions may also have a potential to improve well-being in patients with DM type 2, although the results did not reach statistical significance in favor of the intervention [38], which is in line with our results.

The use of mobile technologies and their innovative apps to address lifestyle change in patients with NCD seems to be in its early days, which can explain our limited findings in other NCDs than DM. However, mHealth interventions have been demonstrated as effective to reduce CVD outcomes, body weight, and BMI and to increase adherence to medical therapy, as well as adherence to nonpharmacologic therapy for patients with CVD [18,39]. Telehealth interventions have been demonstrated as potentially effective interventions to improve outcomes in cancer patients [40]. Apps to support self-management in patients with asthma have been pointed as potential effective [41]. Although apps for lifestyle improvement in patients with DM seems to be ahead compared with the other NCDs, we believe that in a few years more studies will exist for CVD, cancer, and chronic pulmonary diseases as well. We screened many studies evaluating apps for CVD, cancer, and chronic pulmonary diseases in the screening phase of this systematic review; however, most of them were excluded because of follow-up time and outcomes. The reason why apps for DM is major and ahead compared with the other NCDs may be because of difficultness in developing apps that are feasible and with high utility for the more complex NCDs.

App as an intervention can be defined as a complex intervention defined as interventions containing several interacting components [42]. All studies included in his review used apps with several and different components as the main part of the intervention. Most of the studies also had additional support (see Table 2). It can be difficult to understand the cause of any effects of a complex intervention, and therefore, it is crucial to have an idea of the underlying theory of the intervention [42]. In this review, 5 studies [21,22,26,27,30] showed some underlying theory of their intervention. However, it was just one of the included studies that explicitly mentioned their predefined theoretical framework for the intervention [21]. For Internet interventions, it is shown that if a theoretical framework based on several BCTs is incorporated, the interventions are more effective [12]. This may be because different techniques target different stages of a behavioral change process [43]. All studies included in this review used different kinds of feedback and monitoring as BCTs in the app (Table 2). BCTs that have been reported as effective and feasible, especially individualized feedback, have been pointed as being essential to behavioral change and improvement of lifestyle factors [12,44].

Self-management is an important part of the treatment in NCDs. PA, exercise, and a health-promoting diet are the keys to enable a good life while coping with the disease, as well as a possibility to reduce morbidity and mortality [45]. As these are all such important aspects, it is interesting that none of the studies included in this review objectively measured PA or physical fitness.

In conclusion, the results of this study demonstrate that there is limited research of the use of apps for other NCDs than DM with a follow-up of at least 3 months. For DM, the use of apps seems promising to improve lifestyle factors, especially to decrease HbA_1c_. As self-management, including PA and healthy diet, is the key in treatment for all NCDs, it is plausible to believe that such an intervention may also be promising for other NCDs than DM. However, this systematic review clearly indicates a need of further research to evaluate the effect of apps for follow-up for NCDs before implications for practice can be concluded. Especially, there is a need of powered long-term (at least a year) studies for NCDs to be able to evaluate the real effect as NCD patients need to handle their diseases for the rest of their lives. Furthermore, this review reaffirms that future studies must ensure that complex interventions, such as apps, are based on a theoretical framework to bring out the desired behavior change and to understand the impact of the intervention. Finally, appropriate measurements based on the aim of the intervention are always warranted.

## Appendix

Multimedia Appendix 1 Search strategy.

## Abbreviations

BCT: behavioral change technique
BMI: body mass index
CVD: cardiovascular disease
DM: diabetes mellitus
GRADE: Grading of Recommendations Assessment, Development, and Evaluation
HbA_1c_: glycated hemoglobin
mHealth: mobile health
NCD: noncommunicable disease
PA: physical activity
PRISMA: Preferred Reporting Items for Systematic Reviews and Meta-Analyses
QoL: quality of life
WHO: World Health Organization
