# Supplementary material for: The Effectiveness of Smartphone Apps for Lifestyle Improvement in Noncommunicable Diseases: Systematic Review and Meta-Analyses
Source: J Med Internet Res. 2018 May 4;20(5):e162. doi: 10.2196/jmir.9751 (PMC5960039; doi:10.2196/jmir.9751)
Supplement: Multimedia Appendix 1 [file jmir_v20i5e162_app1.pdf]

Systematic search conducted 23th of February 2017.

Embase: (mobile application/ OR (mobile health or smartphone app* or phone app* or mobile app* or cellphone app* or telephone app* or mhealth).tw,kw. AND non insulin dependent diabetes mellitus/ OR cardiovascular disease/ or exp heart disease/ or exp vascular disease/ OR exp neoplasm/ OR exp lung disease/) AND (follow up/ OR (longterm or long-term or follow-up or one-year result* or month* result*).tw,kw.)

Resulted in 192 articles

Medline: (Mobile Applications/ OR (mobile health or smartphone app* or phone app* or mobile app* or cellphone app* or telephone app* or mhealth).tw,kw,kf.) AND (diabetes mellitus, type 2/ or diabetes mellitus, lipoatrophic/ OR cardiovascular diseases/ or exp heart diseases/ or exp vascular diseases/ OR exp Lung Diseases/ OR exp Neoplasms/)

Resulted in 525 articles

Cinahl: ((MH "Mobile Applications") OR TI ((mobile health or smartphone app* or mobile app* or phone app* or cellphone app* or Telephone app* or mhealth)) OR AB ((mobile health or smartphone app* or phone app* or mobile app* or cellphone app* og telephone app* or mhealth)) AND ((MH "Diabetes Mellitus, Type 2") OR (MH "Vascular Diseases+") OR (MH "Heart Diseases+") OR (MH "Cardiovascular Diseases+") OR (MH "Neoplasms+") OR (MH "Lung Diseases+"))

Resulted in 220 articles

Academic Search Premier: TI ((mobile health or smartphone app* or mobile app* or phone app* or cellphone app* or Telephone app* or mhealth)) OR AB ((mobile health or smartphone app* or phone app* or mobile app* or cellphone app* og telephone app* or mhealth)) AND ((TI Diabetes* OR AB Diabetes*) OR (TI (Cancer* or neoplasm*) OR AB (Cancer* or neoplasm*)) OR (TI (pulmonary disease* or lung disease* or respiratory disease*) OR AB (pulmonary disease* or lung disease* or respiratory disease*)) OR (TI (Cardiovascular disease* or coronary heart disease* or vascular disease* or stroke) OR AB (Cardiovascular disease* or coronary heart disease* or vascular disease* or stroke))

Resulted in 560 articles

Cochrane reviews and trials: ([Mobile Applications] OR ((mobile next health or phone next app* or cellphone next app* or telephone next app* or mobile next app* or Smartphone next app* or mhealth):ti,ab,kw)) AND ([Diabetes Mellitus, Type 2] OR [Cardiovascular Diseases] OR [Heart Diseases] OR [Vascular Diseases] OR [Lung Diseases] OR [Neoplasms])

Resulted in 91 articles (3 Cochrane Reviews and 88 trials)
